# Sir Charles Scudamore on Inhalation in Tubercular Phthisis

**Published:** 1834-04-01

**Authors:** 


### SIR CHARLES SCUDAMORE ON INHALATION OF IODINE AND CONIUM IN TUBERCULAR PHTHISIS. Second Edition. 1834.

THIS edition is very considerably altered and improved. The objections which were made to the first edition, of keeping the methodus medendi somewhat secret, and of not stating fully the proportions and preparations of the remedies used by him, are now removed, as the following extract will shew.

" Iodine, mixed with hemlock, constitutes the principal remedy which I have employed.

As, by mixing the tincture of iodine with water, the iodine itself separates into flakes which become precipitated, and as 7000 parts of water are required for its solution, I found it expedient to form a preparation which should be uniform, and preserve its transparency when united with water in any proportions. This admixture is effected by adding together iodine, hydriodate of potash, distilled water, and alcohol. The following is the formula which I prefer on commencing with the treatment of iodine inhalation :

| | | |
|---|---|---|
| ℞. | Iodin. ............ | gr. v. |
| | Potassæ Hydriodat.. | gr. iij. |
| | Aquæ distillat...... | ℥v. |
| | Alcoholis.......... | ʒij. |
| | Tinct. conii*....... | ʒvj.—M. fiat mistura. |

I found it expedient to use the smallest proportion of the hydriodate which would serve the purpose of rendering the iodine soluble, and not enough to engage much of the iodine itself. The addition of the tincture of conium is im-

* "A saturated tincture."

portant; as, together with its distinct operation as a sedative, it softens the action of the iodine; and this property of dimishing the sharpness of the iodine, during the process of inhaling, is more effectually produced by the previous combination of all the ingredients; but the mixture should not be long kept, as in that case the iodine would undergo considerable change, and its power become too much reduced. Also when it is desired to have the iodine solution in the most active state, the conium, if mixed with it at all, should be added only at the time of using the inhalation.

The following are the other medicinal substances which I have used for the purposes of inhalation:

A saturated solution of pure chlorine gas in distilled water.

A saturated tincture of stramonium, prepared from the dried leaves and stalks.

A saturated tincture of belladonna, prepared from the dried leaves.

A saturated tincture of the lobelia inflata.

A spirituous tincture of ipecacuanha, prepared from the roots.

A saturated tincture of digitalis.

Hydrocyanic acid, of the specific gravity ·992.

The pure sulphuric æther.

In the history of my treatment I shall have occasion to describe the manner in which I have employed these medicines." 10.

The following case and observations will complete all that is necessary to be stated respecting the *mode* of using inhalation.

" *Bronchitis, acute, and afterwards chronic; Inhalation, as a Part of the Treatment, proving very decidedly useful.*

A gentleman, aged thirty, of slight form, and not of strong constitution, twice ill with pneumonia within the last three years, was attacked with acute bronchitis, and had been ill a fortnight when I was first consulted. He had been bled once from the arm; a blister had been applied; and medicines had been administered, with relief to the most active part of the disease; but I found him suffering from an assemblage of troublesome symptoms. The pulse was frequent, but free from hardness; there was some heat of skin, and, towards morning, after a restless night, perspiration was always considerable, and sometimes excessive. There was some sense of tightness and oppression of the chest, and the breathing was, by very slight exertion, inconveniently hurried. The cough was irritable both by day and night. The expectoration was partly flaky and yellowish, but chiefly of the mucilaginous kind, and very viscid. The tongue was coated; the bowels, for the most part, confined; the urine deposited lateritious sediment.

In similar cases, in which febrile irritation prevailed, as the attendant of some remaining inflammatory action in the bronchial tubes, I had found advantage from joining digitalis with the other ingredients in the state of herb. I used the following in the present instance:

Of digitalis and conium, cut into fine portions, each ten grains; powdered ipecacuanha two grains; water, of the temperature of 80°, raised by means of a lamp to 130° or 140°, as the patient should find most comfortable. To be used three times a day.

This inhaling mixture produced very good effects; relieving the irritability of the cough, and producing a more easy expectoration. The urine deposited dense mucus very copiously.

I joined other means of treatment, as the use of alteratives and aperients, with salines and diuretics. I applied the acetic acid solution of cantharides to the chest. The regular blistering plaster had lately produced so much inconvenient strangury, that he was glad to be assured that he would avoid such inconvenience by having this preparation substituted.

In four or five days, I changed the inhaling mixture for that of the iodine and conium; and from its use, almost without any further internal medicine, all complaint was removed in about a fortnight. The expectoration was changed by its influence, in a very short time, from the ropy state, which I just now described, to flaky mucus: and which, ere long, differed but little from the thin secretion which belongs to slight irritation of the membrane.

*Observations.*—Although I should avoid all proposal of inhalation during the state of acute bronchitis, yet I am persuaded of the propriety of adopting the treatment, without delay, after the removal of the inflammatory symptoms. In using the tinctures for inhalation, it is to be considered that the proportion of spirit is very small in the dose of the medicine necessary to be employed; and, becoming so largely diluted with water as it is in the inhaler, the alcoholic stimulus can scarcely be reckoned objectionable, when the disadvantage, if any, is counterbalanced by its causing the properties of the medicine to be volatilised the more readily. But, in any case in which the practitioner may think it an objection, he may easily have recourse to several medicines in the state of herb; namely, digitalis, conium, stramonium, belladonna, lobelia inflata, and perhaps some other plants. When convenient, I would choose them in the fresh state; for then I should expect to obtain their volatile principles in the greatest perfection.

In order to use these herbs to most advantage, it is requisite to have an inhaler with a lamp.* The water should be mixed with the ingredients first at the temperature of 80°, and then gradually raised to 130°, 140°, or even higher, exactly according to the feelings of the patient.

In my future experience, I may probably have more occasion to speak of this mode of using the inhaling treatment. I have, on former occasions, employed the saturated tincture of digitalis with the other tinctures, and have been certain that it has had the effect of retarding the pulse and proving useful.

I shall here advert to the remarkable circumstance of the very copious secretion of mucus appearing in the urine, which often takes place on the subsidence of the acute symptoms in bronchitis. The glass, into which the urine is put for inspection, will sometimes exhibit dense mucus almost to the very top. I have regarded such secretion as a curative effort, and as one of the critical indications of the abatement of inflammation." 173.

A considerable number of new cases are added to this edition—several of them important ones. We have tried the inhalation in a few cases of tubercular phthisis, and certainly observed relief from the cough during several hours of the night, after a copious inhalation at bed-time, and this without a succeeding check to the expectoration too often experienced after the exhibition of opiates. Beyond this mitigation of symptoms, our experience does not enable us to say any thing in favour of the remedy.

---

* "Mr. Garden, of Oxford Street, has inhalers of this description, very ingeniously constructed, with a small thermometer to be inserted in the middle tube. For the use of the tinctures, the cheaper and more simple inhaler answers perfectly well. The heat of 120°, or even less, is sufficient to bring off the volatile principles of the fluid preparations."

